# Insurance Coverage and Perinatal Health Care Use Among Low-Income Women in the US, 2015-2017

**DOI:** 10.1001/jamanetworkopen.2020.34549

**Published:** 2021-01-27

**Authors:** Lindsay K. Admon, Jamie R. Daw, Tyler N. A. Winkelman, Katy Backes Kozhimannil, Kara Zivin, Michele Heisler, Vanessa K. Dalton

**Affiliations:** 1 Department of Obstetrics and Gynecology, University of Michigan, Ann Arbor; 2 Department of Health Policy and Management, Columbia University Mailman School of Public Health, New York, New York; 3 Division of General Internal Medicine, Department of Medicine, Hennepin Healthcare, Minneapolis, Minnesota; 4 Division of Health Policy and Management, University of Minnesota School of Public Health, Minneapolis; 5 Department of Psychiatry, University of Michigan, Ann Arbor; 6 Department of Internal Medicine, University of Michigan, Ann Arbor

## Abstract

This cross-sectional study uses 2015-2017 data from the Pregnancy Risk Surveillance and Monitoring System to examine the association between health insurance coverage and use of perinatal health care among low-income women in the US.

## Introduction

A majority of low-income women experience disruptions (gaps and transitions) in health insurance during pregnancy and the first year post partum (the perinatal period).^1,2,3^ We characterized the association between health care use and 4 distinct patterns of insurance coverage across the perinatal period among low-income pregnant and postpartum women.

## Methods

This cross-sectional study used pooled 2015-2017 data from the Pregnancy Risk Surveillance and Monitoring System (PRAMS),^4^ a survey of postpartum women in 40 states and New York City that includes health insurance status at 3 time points: (1) preconception, based on self-reported insurance status 1 month before conception; (2) delivery, based on the primary payer for childbirth; and (3) post partum, based on self-reported insurance status at survey completion (3 to 6 months after childbirth for most women). We limited the sample to women with complete insurance information and household incomes less than 138% of the federal poverty level, the minimum required by federal statute to qualify for pregnancy-related Medicaid.^5^ The University of Michigan institutional review board deemed the study exempt from the need for institutional review board approval and informed consent because it used publicly available, deidentified data. This study followed the Strengthening the Reporting of Observational Studies in Epidemiology (STROBE) reporting guideline.

We hierarchically characterized insurance at each time point into 3 categories: Medicaid, private, or uninsured. We then generated 4 insurance patterns across these time points: continuous insurance, shifts between private and Medicaid coverage, shifts between insurance and uninsurance, and continuous uninsurance. We examined 3 health care use outcomes: prenatal care in the first trimester, in-hospital birth, and postpartum visit attendance. For each insurance pattern, we estimated the probability of each outcome using weighted logistic regression with margins at observed sample values. We weighted all models using PRAMS sample weights and adjusted for age, race/ethnicity, educational level, marital status, and parity. All statistical tests were 2-sided, and *P* < .05 was considered statistically significant. Statistical analyses were conducted from December 27, 2019, to November 19, 2020, using Stata, version 14.2 (StataCorp LLC).

## Results

The study sample consisted of 39 378 women with a mean (SD) age of 27.4 (5.9) years. Of these, 43.6% were continuously insured, 21.3% experienced shifts between private coverage and Medicaid, 32.8% experienced shifts between insurance and uninsurance, and 2.4% were continuously uninsured. Spanish-speaking Hispanic women composed 60.1% (95% CI, 52.9%-66.8%) of the continuously uninsured category (Table). Women who experienced a shift between insurance and uninsurance or continuous uninsurance were significantly less likely to receive prenatal care in the first trimester (marginal differences, −12.5% [95% CI, –14.8% to 10.2%] and −24.2% [95% CI, –30.8% to –17.6%], respectively) or a postpartum visit (marginal differences, −2.1% [95% CI, –3.7% to –0.5%] and −9.6% [95% CI, –15.9% to –3.4%], respectively) compared with those who had continuous insurance (adjusted probabilities, 77.8% [95% CI, 76.8%-78.8%] and 84.3% [95% CI, 83.4%-85.2%], respectively; *P* < .001 for each comparison) (Figure). Almost all women (>98.4%) across the first 3 categories of insurance coverage experienced in-hospital births, whereas 80.0% of women with continuous uninsurance experienced an in-hospital birth (marginal difference compared with continuous insurance, −19.4%; 95% CI, −24.5 to −14.2; *P* < .001).

**Table.** Characteristics of Respondents by Insurance Coverage Pattern From the Pregnancy Risk Assessment Monitoring System 2015-2017^a^

Characteristic	Continuous insurance (n = 19 510)	Private-Medicaid discontinuity (n = 8263)	Insured-uninsured discontinuity (n = 10 909)	Continuous uninsurance (n = 696)
Age, y				
≤19	10.7 (9.9-11.5)	10.2 (9.1-11.4)	6.4 (5.6-7.4)	6.1 (3.1-11.6)
20-24	29.9 (28.8-31.0)	31.6 (30.0-33.3)	30.6 (28.9-32.3)	23.0 (16.6-31.0)
25-29	30.4 (29.3-31.5)	29.4 (27.7-31.1)	30.0 (28.4-31.6)	26.0 (20.3-32.6)
30-34	18.2 (17.3-19.1)	17.9 (16.6-19.2)	20.5 (19.2-22.0)	23.4 (18.5-29.2)
≥35	10.8 (10.1-11.5)	11.1 (9.9-12.3)	12.5 (11.4-13.8)	21.6 (16.3-28.0)
Race/ethnicity				
White, non-Hispanic	47.1 (46.0-48.2)	43.3 (41.5-45.0)	31.4 (30.1-32.8)	18.5 (14.4-23.6)
Black, non-Hispanic	23.9 (23.0-24.8)	24.2 (22.8-25.7)	14.1 (13.1-15.1)	3.4 (2.0-5.8)
Hispanic, Spanish speaking	6.3 (5.7-7.0)	9.2 (8.1-10.5)	29.8 (28.2-31.5)	60.1 (52.9-66.8)
Hispanic, English speaking	11.9 (11.1-12.7)	13.3 (11.9-14.8)	17.0 (15.5-18.6)	12.8 (8.0-19.8)
Asian or Pacific Islander	4.1 (3.7-4.5)	3.9 (3.3-4.5)	3.1 (2.6-3.7)	1.5 (0.7-3.4)
Indigenous	1.4 (1.3-1.6)	0.9 (0.8-1.1)	1.4 (1.3-1.6)	0.7 (0.3-1.7)
Other or mixed race/ethnicity^b^	3.7 (3.3-4.1)	4.0 (3.4-4.8)	2.3 (2.0-2.7)	2.0 (0.8-5.0)
Missing or unknown race/ethnicity	1.6 (1.4-2.0)	1.2 (0.9-1.6)	0.9 (0.7-1.2)	1.1 (0.4-2.7)
Educational level				
Less than high school	20.8 (19.8-21.8)	19.4 (18.0-20.9)	30.3 (28.6-32.0)	53.2 (45.8-60.5)
High school	39.8 (38.7-41.0)	40.8 (39.0-42.7)	37.3 (35.6-39.1)	31.2 (24.5-38.9)
More than high school	38.5 (37.4-39.6)	39.2 (37.5-41.0)	31.6 (30.0-33.2)	15.1 (11.1-20.2)
Marital status				
Not married	65.4 (64.3-66.5)	66.9 (65.2-68.5)	60.9 (59.3-62.5)	45.9 (38.7-53.3)
Married	34.5 (33.4-35.6)	33.0 (31.4-34.7)	39.0 (37.4-40.6)	54.1 (46.7-61.3)
Parity				
Primiparous	27.7 (26.7-28.8)	35.5 (33.8-37.3)	31.4 (29.7-33.0)	21.2 (15.6-28.2)
Multiparous	72.1 (71.0-73.2)	64.2 (62.5-66.0)	68.4 (66.7-70.0)	78.7 (71.8-84.4)

^a^ Data are presented as unweighted numbers (95% CI) or weighted proportions (95% CI).

^b^ Other or mixed race/ethnicity was included as a self-identification category in the Pregnancy Risk Assessment Monitoring System survey.

**Figure.**
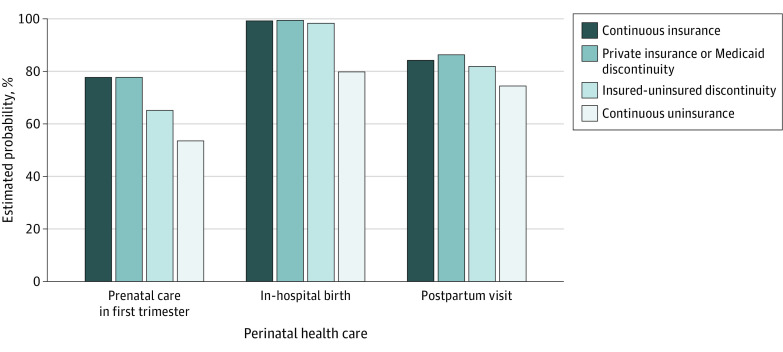
Perinatal Health Care Use by Insurance Coverage Pattern Among Low-Income Women Data are from the Pregnancy Risk Assessment Monitoring System, 2015-2017 (N = 39 378) and are presented as weighted proportions.

## Discussion

Continuous uninsurance or shifts between insurance and uninsurance from preconception to postpartum were associated with a lower likelihood that women received early prenatal care or recommended postpartum care compared with women with continuous insurance. Hispanic, Spanish-speaking women composed nearly two-thirds of the women with continuous uninsurance, suggesting particularly high barriers to enrollment among this population potentially associated with immigration status. Study limitations include the use of self-reported insurance status, exclusion of 10 states that did not participate in PRAMS, and the inability to discern citizenship, which may affect insurance eligibility. Improving receipt of recommended maternity care among women who experience uninsurance in the perinatal period is a clinical and policy priority. Adoption of the Patient Protection and Affordable Care Act’s Medicaid expansion has improved perinatal insurance continuity for low-income women.^3^ National efforts to extend Medicaid through the first year post partum are supported by the American Medical Association and are another possible mechanism for improving health care access among postpartum women.^6^
